# Characterization of neuroendocrine regulation- and metabolism-associated molecular features and prognostic indicators with aid to clinical chemotherapy and immunotherapy of patients with pancreatic cancer

**DOI:** 10.3389/fendo.2022.1078424

**Published:** 2023-01-20

**Authors:** Biao Zhang, Qihang Yuan, Bolin Zhang, Shuang Li, Zhizhou Wang, Hangyu Liu, Fanyue Meng, Xu Chen, Dong Shang

**Affiliations:** ^1^ Department of General Surgery, Clinical Laboratory of Integrative Medicine, The First Affiliated Hospital of Dalian Medical University, Dalian, Liaoning, China; ^2^ Department of Visceral, Vascular and Endocrine Surgery, Martin-Luther-University Halle-Wittenberg, University Medical Center Halle, Halle, Germany

**Keywords:** pancreatic cancer, neuroendocrine regulation, metabolism, prognosis, immune, mutation, drug sensitivity

## Abstract

The worldwide prevalence of pancreatic cancer has been rising in recent decades, and its prognosis has not improved much. The imbalance of substance and energy metabolism in tumour cells is among the primary causes of tumour formation and occurrence, which is often controlled by the neuroendocrine system. We applied Cox and LASSO regression analysis to develop a neuroendocrine regulation- and metabolism-related prognostic risk score model with three genes (GSK3B, IL18 and VEGFA) for pancreatic cancer. TCGA dataset served as the training and internal validation sets, and GSE28735, GSE62452 and GSE57495 were designated as external validation sets. Patients classified as the low-risk population (category, group) exhibited considerably improved survival duration in contrast with those classified as the high-risk population, as determined by the Kaplan-Meier curve. Then, we combined all the samples, and divided them into three clusters using unsupervised clustering analysis. Unsupervised clustering, t-distributed stochastic neighbor embedding (t-SNE), and principal component analysis (PCA) were further utilized to demonstrate the reliability of the prognostic model. Moreover, the risk score was shown to independently function as a predictor of pancreatic cancer in both univariate and multivariate Cox regression analyses. The results of gene set enrichment analysis (GSEA) illustrated that the low-risk population was predominantly enriched in immune-associated pathways. “ESTIMATE” algorithm, single-sample GSEA (ssGSEA) and the Tumor Immune Estimation Resource (TIMER) database showed immune infiltration ratings were enhanced in the low-risk category in contrast with the high-risk group. Tumour immune dysfunction and exclusion (TIDE) database predicted that immunotherapy for pancreatic cancer may be more successful in the high-risk than in the low-risk population. Mutation analysis illustrated a positive link between the tumour mutation burden and risk score. Drug sensitivity analysis identified 44 sensitive drugs in the high- and low-risk population. GSK3B expression was negatively correlated with Oxaliplatin, and IL18 expression was negatively correlated with Paclitaxel. Lastly, we analyzed and verified gene expression at RNA and protein levels based on GENPIA platform, HPA database and quantitative real-time PCR. In short, we developed a neuroendocrine regulation- and metabolism-associated prognostic model for pancreatic cancer that takes into account the immunological microenvironment and drug sensitivity.

## Introduction

Pancreatic cancer is becoming an increasingly major health problem throughout the world, with the age-standardized incidence rates rising from 5.0/100,000 persons in 1990 to 5.7/100,000 in 2017 (1). Pancreatic cancer is projected to overtake lung cancer as the major cancer killer in the United States by 2030, moving up from its current position as the third leading contributor to cancer-associated deaths (2–4). Surgery is the only current hope for curing pancreatic cancer, which is exceedingly aggressive and has a very dismal prognosis. However, about 80-85% of patients with pancreatic cancer are diagnosed as unresectable or metastatic due to the occult onset and lack of early screening methods. Even when diagnosed to be resectable, pancreatic cancer prognosis is dismal, with just a 20% 5-year survival probability after surgery (5). Surgery and chemotherapy are common treatment options for pancreatic cancer, however, they have limited effectiveness. Researchers discovered that the median survival periods for individuals with metastatic pancreatic cancer treated with FOLFIRINOX (leucovorin, irinotecan, oxaliplatin, and fluorouracil) or gemcitabine were 11.1 months and 6.8 months, respectively, while those with resected pancreatic cancer treated with modified FOLFIRINOX or gemcitabine had median survivals of 54.4 months and 35.0 months, respectively (6, 7). Although the survival benefit of FOLFIRINOX is superior to gemcitabine, there are also more toxic side effects. Targeted therapy and immunotherapy have made enormous strides in recent years, bringing revolutionary advances to the treatment of cancer, but their effectiveness is not optimal for pancreatic cancer. A study by Hong et al. (8) illustrated that the overall response rate (complete or partial response) of ibrutinib plus durvalumab (a PD-L1-targeting antibody) in the treatment of pancreatic cancer was only 2%. Therefore, it is still extremely vital and urgent to investigate the mechanism of occurrence and development and to identify an effective therapy for pancreatic cancer.

Metabolic alteration is an important topic in cancer biology research, and metabolic reprogramming is considered to be one of the hallmarks of cancer, participating in the process of cancer occurrence, development and metastasis (9). To adapt to the microenvironment of hypoxia and nutrient deficiency, establish survival advantages and achieve rapid growth, tumour cells change their material and energy metabolism patterns, which is called metabolic reprogramming (10). The research found that tumour cells significantly increase the demand and uptake of glucose, rapidly produce ATP through the glycolysis pathway, and aerobic glycolysis is performed even in the presence of oxygen, also referred to as the “Warburg effect”. Lactic acid produced by glycolysis will accumulate in the tumour microenvironment (TME), boost tumour cells invasiveness and reduce the anti-tumour immunity (11). Glutamine is an important source of nitrogen and carbon in biosynthetic reactions. Normal cells can synthesize glutamine by themselves, while tumour cells can obtain glutamine from the microenvironment by solute carrier group in addition to their own synthesis to meet the proliferation needs (12). Lipids, mainly including fatty acids, cholesterol, phospholipids and acrylamide, are not only the basic structure of cell membranes, but also a source of signalling molecules and energy, and the lipid metabolism reprogramming of tumour cells can promote their proliferation, invasion and metastasis (13, 14). Ringel et al. (15) showed that obesity can cause the metabolic changes for fatty acid, impair the function and infiltration for CD8+T cells, and thus inhibit anti-tumour immunity. Hypoxia and nutrient deprivation in TME will lead to metabolic competition between immune and tumour cells, and the high metabolism and strong adaptability of tumour cells will further change the metabolic characteristics of the TME, causing metabolic pressure on immune cells, while continuously accumulating toxic metabolites, negatively affecting the immunity, thereby promoting immune suppression and escape (16, 17). Besides, metabolic alteration in tumour cells is also intimately linked to the sensitivity of chemotherapy, targeted therapy, immunotherapy, and radiotherapy (18–22). Obesity has been shown to increase tumour cells’ resistance to chemotherapy, radiation, and biological and endocrine-targeted treatments by altering the way fatty acids are metabolized in TME (23).

The imbalance of material and energy metabolism of tumour cells is often regulated by the neuroendocrine system. Therefore, tumorigenesis and progression are aided by neuroendocrine control. Cancers of the liver, pancreas, colorectal, breast, and uterus are all thought to be linked to neuroendocrine regulation disorders such as diabetes, obesity, and depression (24–28). An analysis of data from a large-scale cohort study in the US involving 112,818 women and 46,207 men found that those with new-onset diabetes exhibited a 2.97-fold (95% CI, 2.31-3.82) greater risk of developing pancreatic cancer than those without diabetes, while those with long-term diabetes recorded a 2.16-fold (95% CI, 1.78-2.60) higher risk (29). As a digestive organ, the pancreas has both endocrine and exocrine functions, and its lesions are often accompanied by abnormal regulation of blood glucose. Therefore, diabetes is not only a risk factor for pancreatic cancer but also one of its secondary diseases. The mechanism for diabetes causing pancreatic cancer is complex, including hyperglycemia, hyperinsulinemia, insulin resistance, chronic inflammation, and so on (30). Persons with a body mass index (BMI) of 25 to 29.9 were found to have an odds ratio (OR) for pancreatic cancer that was 1.19 (95% CI, 1.02-1.40), whereas those with a BMI of 30 to 34.9 were found to have an OR of 1.62 (95% CI, 1.19-2.21) compared with normal-weight individuals (31). A study by Rebours et al. (32) found that pancreatic fatty infiltration was related to the formation of pancreatic intraepithelial neoplasia.

Based on the above evidence, our study was the first to develop a neuroendocrine regulation- and metabolism-related prognostic model of pancreatic cancer utilizing The Cancer Genome Atlas (TCGA) database, then verified the model through Gene Expression Omnibus (GEO) database. Unsupervised clustering, t-distributed stochastic neighbor embedding (t-SNE), and principal component analysis (PCA) were further conducted to demonstrate the consistency and reliability for the prognostic model. In addition, the association between prognostic models and clinicopathological characteristics, the tumour immune microenvironment, the tumour mutation load, and treatment sensitivity was investigated. Finally, we explored and verified gene expression at RNA and protein levels based on GENPIA platform, the Human Protein Atlas (HPA) database and quantitative real-time PCR, and explored gene expression distribution in different subcellular structures and cell types *via* HPA database.

## Materials and methods

### Data acquisition and processing

The transcriptome profiles (including 178 pancreatic tumour tissues and 4 normal pancreatic tissues), somatic mutation status, copy number variation (CNV), and matching clinical data (including 185 pancreatic cancer samples) were extracted in the TCGA database (https://portal.gdc.cancer.gov/). To get the validation set, GSE28735 (including 45 pancreatic cancer samples), GSE62452 (including 69 pancreatic cancer samples) and GSE57495 (including 63 pancreatic cancer samples) were derived in the GEO database (https://www.ncbi.nlm.nih.gov/geo/). The “SVA” R package was utilized to eliminate any batch effects that existed across various data sets (33). To elucidate the differences between pancreatic tumors and normal samples more precisely, the TCGA dataset (containing 178 pancreatic tumour tissues and 4 normal tissues) and the Genotype-Tissue Expression Project (GTEx) project dataset (containing 167 normal pancreatic tissues) were extracted in the UCSC Xena database (https://xenabrowser.ucsc.edu/datapages/) and the gene expression data were normalized using the log2(x+1) transformation.

### Identification of differentially-expressed neuroendocrine regulation- and metabolism-related genes

Based on the “limma” R packages, with the filter condition: |log2FC| > 1, and adjusted p-values < 0.05, we detected differentially expressed genes (DEGs) between tumour and normal samples. The screened differential genes were visualized by the “ggplot2” R package. Neuroendocrine regulation-related and metabolism-related genes were retrieved in the GeneCards database (https://www.genecards.org/) (33), setting filter parameters: relevance score > 5. We identified differentially expressed neuroendocrine regulation- and metabolism-related genes (NMRGs) by merging DEGs and NMRGs.

### Development and analysis of protein-protein interaction network

We explored the possible interactions for differentially expressed NMRGs using the STRING database (https://cn.string-db.org/) at a minimum interaction score of 0.4 (medium confidence) (34). The PPI network was created and shown utilizing Cytoscape (version 3.9.1). The PPI network’s critical modules and hub genes were then isolated utilizing the MCODE plugin, with screening conditions established as below: Max depth = 100, k-score = 2, node score cutoff = 0.2, and degree cutoff = 2.

### Genetic alteration and enrichment analysis

Mutation landscape of hub genes in pancreatic cancer was analyzed and visualized utilizing the “maftools” R package. The “RCircos” R programme was applied to evaluate and illustrate the CNV of hub genes. “org.Hs.eg.db”, “clusterProfiler”, “enrichplot” and “ggplot2” R packages were applied to perform Gene Ontology (GO) and Kyoto Encyclopedia of Genes and Genomes (KEGG) enrichment analyses for the above differentially expressed NMRGs, based on the filter condition: q value < 0.05. GO enrichment analysis involves molecular function (MF), cellular component (CC), and biological process (BP).

### Construction and verification of the prognostic model

Patients from TCGA were randomized at a ratio of 7:3 into the training and internal validation sets by utilizing the “caret” R package. The external validation set included patients from GSE28735, GSE62452, and GSE57495. Firstly, we conducted univariate Cox regression analysis to preliminarily filter the genes linked to prognosis. Next, the least absolute shrinkage and selection operator (LASSO) regression analysis was utilized to overcome overfitting with the “glmnet” R package. Lastly, a predictive model was developed utilizing multivariate Cox regression analysis. Below is the risk score equation for the prognostic model:


$$Risk\;score=\sum_{1}^{n}\text{Coefficient }\left({\text{RNA}}_{i}\right)\times \text{ Expression}\left({\text{RNA}}_{i}\right)$$

Patients were assigned a risk score using the approach and then categorized into high- and low-risk groups as per how their score compares to the training set’s median risk score. To contrast the prognosis between the high- and low-risk groups, survival curves were generated utilizing Kaplan-Meier method. The prognostic model was evaluated utilizing a time-dependent Receiver Operating Characteristic (ROC) curve and area under the curve (AUC).

### Cluster analysis

For identifying the prognostic model’s consistency and dependability, we pooled patients from TCGA, GSE28735, GSE62452, and GSE57495 and performed an unsupervised cluster analysis using the “ConsensusClusterPlus” R package. The cumulative distribution function (CDF) curve and consensus matrix were applied to get the best possible clustering parameter values. The Kaplan-Meier technique was utilized to compare survival times across the categories. Additional verification of the clusters and prognostic model was achieved *via* the use of PCA and t-SNE.

### Clinicopathologic correlation, independent prognostic analysis, and nomogram model construction

We integrated patients’ clinical data and risk scores categorized them as per the clinicopathological criteria, followed by performing the Wilcoxon and Kruskal-Wallis rank sum test to assess risk scores across groups. For identifying whether the risk score independently acted as a predictor of pancreatic cancer, univariate and multivariate Cox regression analyses were conducted. Clinicopathological parameters (age, stage, pathological grade, and gender) and risk scores were considered in the development of the nomogram model for predicting the prognosis of pancreatic cancer utilizing the “rms” R package. The predictive performance of the nomogram model was tested using the calibration curve (35).

### Gene set enrichment analyses

To compare the variations in biological processes and metabolic pathways between low- and high-risk groups, gene set enrichment analyses (GSEA) based on gene sets “c5.go.v7.5.1.symbols.gmt” and “c2.cp.kegg.v7.5.1.symbols.gmt” was executed utilizing R package “limma”, “org.hs.eg.db”, “ClusterProfiler” and “enrichplot”, the filter criteria was set as follows: |normalized enrichment score (NES)| > 1 and q value < 0.05.

### Immune analysis

To evaluate the variations in TME between low- and high-risk groups, the “ESTIMATE” algorithm was utilized to compute each patient’s stromal, immune, and ESTIMATE scores (36). Single-sample GSEA (ssGSEA) was carried out to determine the infiltration scores of 16 distinct immune cells and the activity score of 13 immune-associated pathways for each patient utilizing the “GSVA” and “GSEABase” R packages. Immunocyte infiltration scores of all TCGA tumors were retrieved from the Tumour Immune Estimation Resource database (TIMER, http://timer.cistrome.org/) to further evaluate their association with risk scores. These scores were calculated using a variety of algorithms, notably, MCPCOUNTER, EPIC, QUANTISEQ, XCELL, CIBERSORTABS, TIMER, and CIBERSORT (37). Tumour immune dysfunction and exclusion (TIDE) can measure the sensitivity of immune checkpoint blockade by simulating tumour immune evasion mechanism (38). The online TIDE platform (http://tide.dfci.harvard.edu/) was utilized to determine the TIDE score, as well as T-cell dysfunction and exclusion scores for each patient.

### Mutation analysis

To compare and contrast the mutation profiles of individuals at high and low risk, we utilized the “maftools” in R package. Somatic, insertion, base substitution, coding, and deletion mutations were all included in the definition of tumour mutation burden (TMB) (39). TMB estimates are calculated as overall mutation frequency divided by 38 Mb since this value is commonly retrieved based on the length of human exons (40). The association between risk scores and TMB was analyzed with the use of the Spearman correlation test. The ideal cut-off value of TMB was also determined, and the affect of TMB on the prognosis of pancreatic cancer was assessed, with the use of the “survminer” and “survival” R packages.

### Drug sensitivity analysis

To analyze the disparity in medication responsiveness between high- and low-risk groups, we used the “pRRophetic” package in R (41). Using the CellMiner database (https://discover.nci.nih.gov/cellminer/), we retrieved pertinent gene expression data and Food and Drug Administration (FDA) authorized drug sensitivity data to further assess the link between drug sensitivity and the genes in the predictive model (42). The connection between gene expression and drug responsiveness was analyzed using the Pearson test.

### Gene expression and distribution exploration

Differences in the expression of GSK3B, IL18 and VEGFA at the RNA level between normal and tumor pancreatic tissues were analyzed *via* the GEPIA platform (http://gepia2.cancer-pku.cn/#analysis). Protein expression patterns in normal cells, tissues, as well as cancer tissues may be generated using the public HPA platform (https://www.proteinatlas.org/) (43). For protein-level confirmation of gene expression, we used immunohistochemistry pictures of normal and pancreatic cancer tissue obtained from the HPA database. We also obtained relevant data and photos to examine the distribution of gene expression in diverse subcellular structures and cell types. In contrast to traditional bulk RNA-seq that generates mixed gene expression data from tissues, single cell RNA-seq can provide a transcriptional data of individual cells (44). Tumor Immune Single-cell Hub (TISCH, http://tisch.comp-genomics.org) is a single cell RNA-seq database focusing on TME (45). The distribution of model genes in different cells in the TME of pancreatic cancer was further explored through the TISCH database.

### Cell lines and culture

Cell lines of HPDE6-C7, CF-PAC1, Panc-1 and BxPC-3 were from our laboratory, which were consistent with our previous research. And the HPDE6-C7 was a human pancreatic ductal epithelium, the others were pancreatic cancer cell lines. We used the with DMEM mixed with 10% FBS (Gibco, USA) to culture HPDE6-C7, BxPC-3, and Panc-1 cell lines. And CF-PAC1 were cultured with IMDM mixed with 10% (FBS) (Procell, China). All the cell lines were incubated at Cell Incubator.

### The validation of the hub RNA expression with quantitative real-time PCR

The total RNAs was isolated from HPDE6-C7 cell lines and CF-PAC1, Panc-1and BxPC-3 cell lines by extraction tool named TRIzol (Accurate Biotechnology). We utilized the Reverse Transcription Reagent to prepare the cDNAs. RT-PCR was carried out by using qPCR Kit (Accurate Biotechnology). The experiment reagents were from our laboratory. And the GAPDH was served as the control standard. The analysis and quantification of RNA expression level adopted the ΔΔCt method. All the primer sequences obtained from GenePharma (Suzhou, China) were for human, which were as follows: IL18, 5’- TCTTCATTGACCAAGGAAATCGG-3’ (Forward), 5’- TCCGGGGTGCATTATCTCTAC-3’ (Reverse); GSK3B, 5’- GCCCAGAACCACCTCCTTTGC-3’ (Forward), 5’- CACCTTGCTGCCGTCCTTGTC-3’ (Reverse); VEGFA, 5’- GCCTTGCCTTGCTGCTCTACC-3’ (Forward), 5’- CTTCGTGATGATTCTGCCCTCCTC-3’ (Reverse).

### Statistical analysis

For this project, we utilized R (version 4.1.2) and GraphPad Prism 9 to conduct statistical analysis and visual representation of data. The Wilcoxon rank sum test was conducted to examine the disparities between the two groups. More than two groups were compared using the Kruskal-Wallis rank sum test. Parametric and nonparametric variables were compared using Pearson or Spearman correlations, respectively. Kaplan-Meier with log-rank test was utilized to analyze survival data. p-value < 0.05 denotes a remarkably outcome.

## Results

### Detection and analysis of differentially-expressed NMRGs

**Figure 1:**
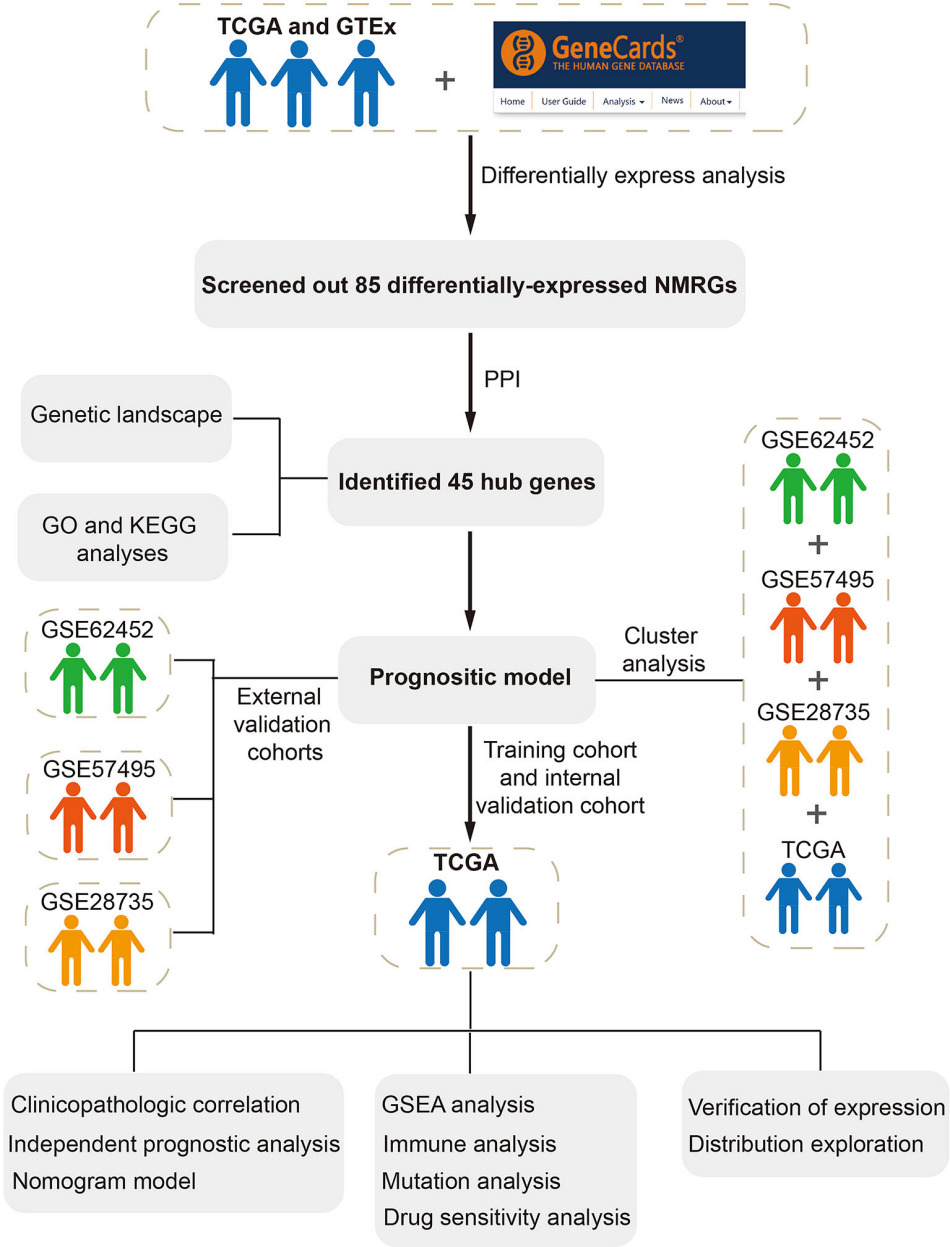
Flowchart in this study.

**Figure 2:**
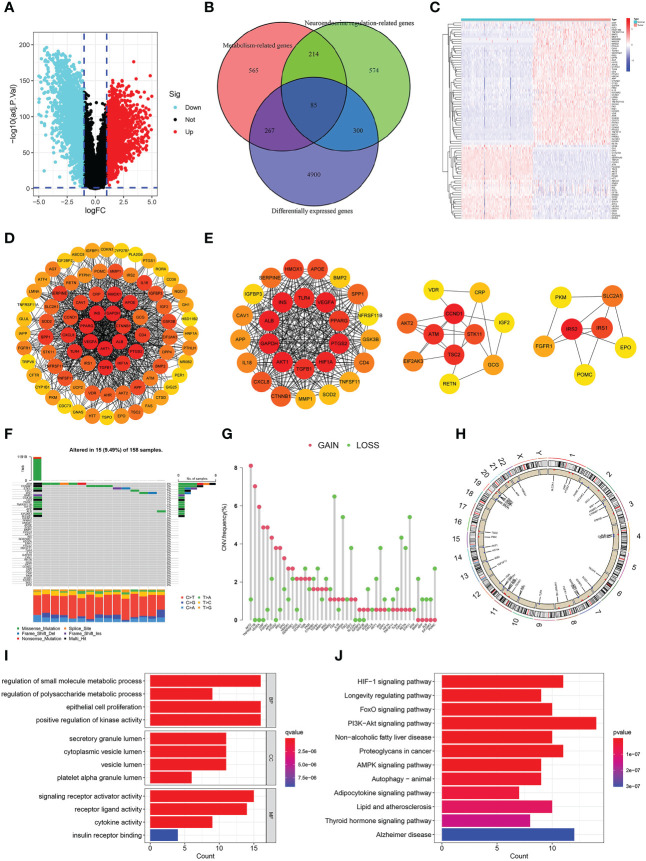
Identification and analysis of differentially-expressed neuroendocrine regulation- and metabolism-related genes (NMRGs). **(A)** A volcano map of differentially expressed genes. **(B)** A venn diagram of intersection of differentially expressed, neuroendocrine regulation-related and metabolism-related genes. **(C)** A heat map of differentially-expressed NMRGs. **(D)** PPI of differentially-expressed NMRGs. **(E)** Three core modules in PPI. **(F)** Genetic mutation of hub genes. **(G)** Frequencies of CNV gain and loss. **(H)** Location of the CNV on the chromosomes. **(I)** GO enrichment analysis. **(J)** KEGG enrichment analysis.

The whole study process is depicted in **Figure 1**. We identified 5552 DEGs between pancreatic tumors and normal samples by combining TCGA and GTEx databases (**Figure 2A**), 1173 neuroendocrine regulation-related genes and 1131 metabolism-related genes from GeneCards database (**Table S1**). In total, 85 differentially-expressed NMRGs were detected by taking intersection (**Figures 2B, C**). We constructed PPI using 85 differentially expressed NMRGs, and further identified three core modules with 45 hub genes using the MCODE plug-in (**Figures 2D, E**). 45 hub genes were preserved for subsequent analysis.

### Genetic landscape and enrichment analysis

We analyzed 45 hub genes for mutation landscape and CNV in pancreatic cancer. The results showed that 15 (9.49%) of 158 samples had gene mutations, and the three most common mutated genes were ATM (4%), CTNNB1 (3%) and SKT11 (2%), missense mutation was the most common type, C>T accounted for the highest proportion in single nucleotide variants (SNV) (**Figure 2F**). CNV was present in all 45 hub genes, and the three most frequent genes are AKT2, TNFRSF11B and VEGFA (**Figure 2G**). **Figure 2H** depicted the chromosomal position of the CNV for each of the 45 hub genes.

We conducted GO and KEGG enrichment analyses on these 45 hub genes to delve into their biological roles and mechanisms. GO enrichment analysis illustrated that “regulation of small molecule metabolic process”, “epithelial cell proliferation”, “Positive regulation of kinase activity” and “regulation of polysaccharide process” were the most remarkably enriched pathways in BP, “secretory granule lumen”, “cytoplasmic vesicle lumen”, “vesicle lumen” and “platelet alpha granule lumen” were the most significantly enriched pathway in CC, “signalling receptor activator activity”, “receptor ligand activity”, “cytokine activity” and “insulin receptor binding” were the most remarkably enriched pathway in MF (**Figure 2I**). KEGG enrichment analysis illustrated that “PI3K−Akt signalling pathway”, “Alzheimer’s disease”, “HIF−1 signalling pathway”, “proteoglycans in cancer”, “non−alcoholic fatty liver disease”, and “thyroid hormone signalling pathway” were the most significantly enriched pathway (**Figure 2J**). We could find that the enriched pathways are mainly related to metabolism, neuroendocrine system diseases and tumors.

### Establishment and validation of the NMRGs-related prognostic model

**Figure 3:**
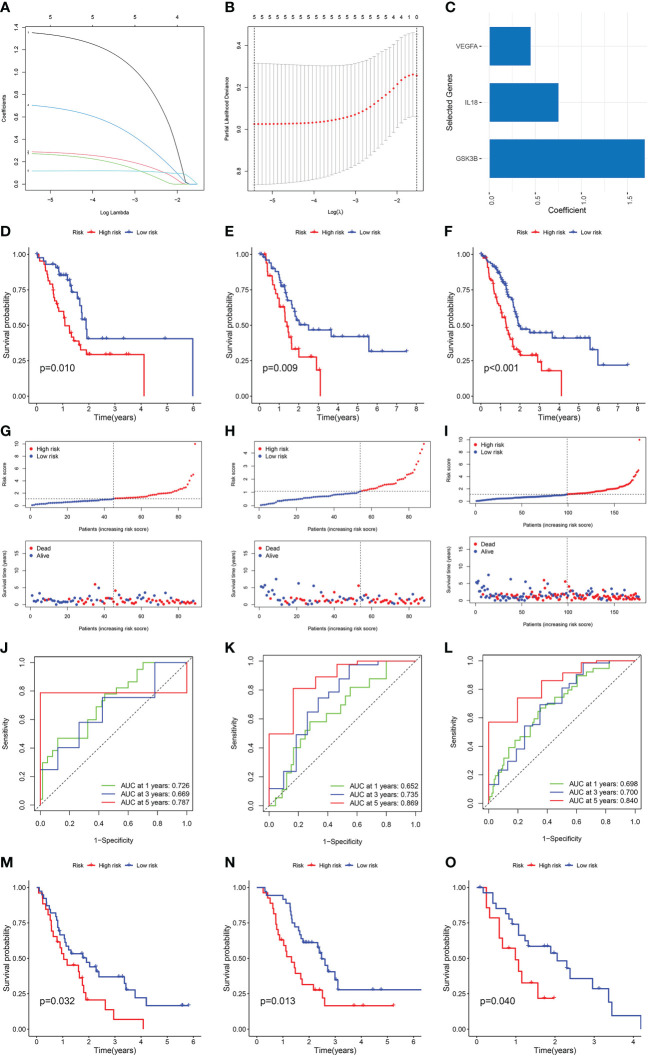
Construction and verification of the NMRGs-related prognostic model. **(A)** LASSO regression analysis with coefficient path diagram. **(B)** LASSO regression analysis with cross-validation curve. **(C)** Coefficient of three genes in the prognostic model. Kaplan-Meier curve of the training set **(D)**, internal validation set **(E)** and the whole TCGA dataset **(F)**. Risk score and survival status distribution of training set **(G)**, internal validation set **(H)** and the whole TCGA dataset **(I)**. ROC curve of the training set **(J)**, internal validation set **(K)** and the whole TCGA dataset **(L)**. Kaplan-Meier survival curve of GSE62452 **(M)**, GSE57495 **(N)** and GSE28735 dataset **(O)**.

Patients from the TCGA cohort were randomised at a 7:3 ratio into the training and internal validation sets. Cox and LASSO regression analyses were used to establish a prognostic model with three NMRGs-related genes (**Figures 3A-C**). Below is the equation of the prognostic model: risk score = (1.69076150270978 * GSK3B expression) + (0.755709134258276 * IL18 expression) + (0.453448880701734 * VEGFA expression). The patient’s survival time in the low-risk group was considerably elevated as opposed to that of patients in the high-risk group, as shown by a survival analysis conducted on the training set, the internal validation set, and the whole TCGA dataset (**Figures 3D-F**). In comparison to the low-risk category, patients classified as the high-risk category fared worse in terms of overall survival (OS) (**Figures 3G-I**). The AUC value for 1, 3 and 5 years were 0.726, 0.669 and 0.787 in the training cohort (**Figure 3J**), 0.652, 0.735 and 0.869 in the internal validation cohort (**Figure 3K**), and 0.698, 0.700 and 0.840 in the whole TCGA cohort (**Figure 3L**), respectively. All of these pointed to the high predictive power of our prognostic model. We used GSE62452, GSE57495 and GSE28735 datasets as external validation sets to additionally illustrate the predictive significance of our prognostic model. Longer survival rates were recorded for patients classified in the low-risk category (**Figures 3M-O**).

### NMRG-based consensus clustering

**Figure 4:**
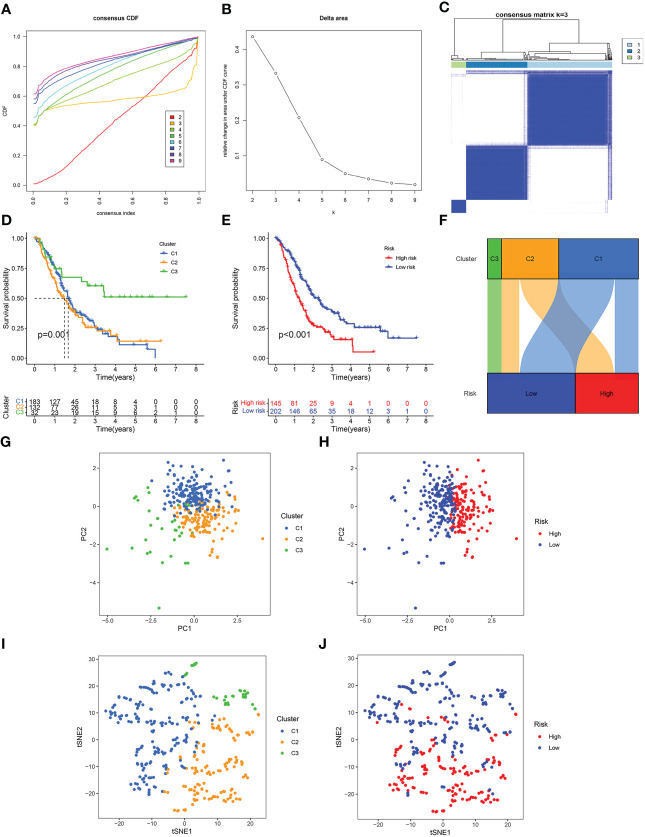
Consensus clustering. **(A)** Cumulative distribution function (CDF) curve. **(B)** Relative change of the area under the CDF curve when cluster number k = 2–9, and the optimal k = 3. **(C)** Heat map of consensus matrix when k = 3. **(D)** Kaplan-Meier curve of three clusters. **(E)** Kaplan-Meier curve of the high- and low-risk score groups. **(F)** Alluvial diagram of changes in three clusters and two risk score groups. **(G)** PCA analysis of three clusters. **(H)** PCA analysis of the high- and low-risk score groups. **(I)** t-SNE analysis of three clusters. **(J)** t-SNE analysis of the high- and low-risk score groups.

To additionally illustrate the reliability of our prognostic model, we combined all the samples from TCGA, GSE62452, GSE57495 and GSE28735, and then divided them into three clusters using unsupervised clustering analysis (**Figures 4A-C**). Survival analysis showed that the survival time of Cluster 3 was remarkably higher than Cluster 1 and Cluster 2 (**Figure 4D**). In all the pooled datasets, the patients having a low risk had a remarkably more favorable prognosis in contrast with those identified as having a high risk (**Figure 4E**). The alluvial diagram displayed the patients’ distribution in the three NMRG-related clusters and two NMRG-related risk score groups, and all cluster 3 patients were mapped to the low-risk subgroup, and all high-risk patients were mapped to cluster 1 and cluster 3, which indicated that our clusters and groups were reasonable and reliable (**Figure 4F**). PCA and t-SNE results showed that our clusters and groups could clearly distinguish different patients, and this further demonstrates the good consistency and reliability of our prognostic model (**Figures 4G-J**).

### Clinicopathologic correlation, independent prognostic analysis, and nomogram model construction

**Figure 5:**
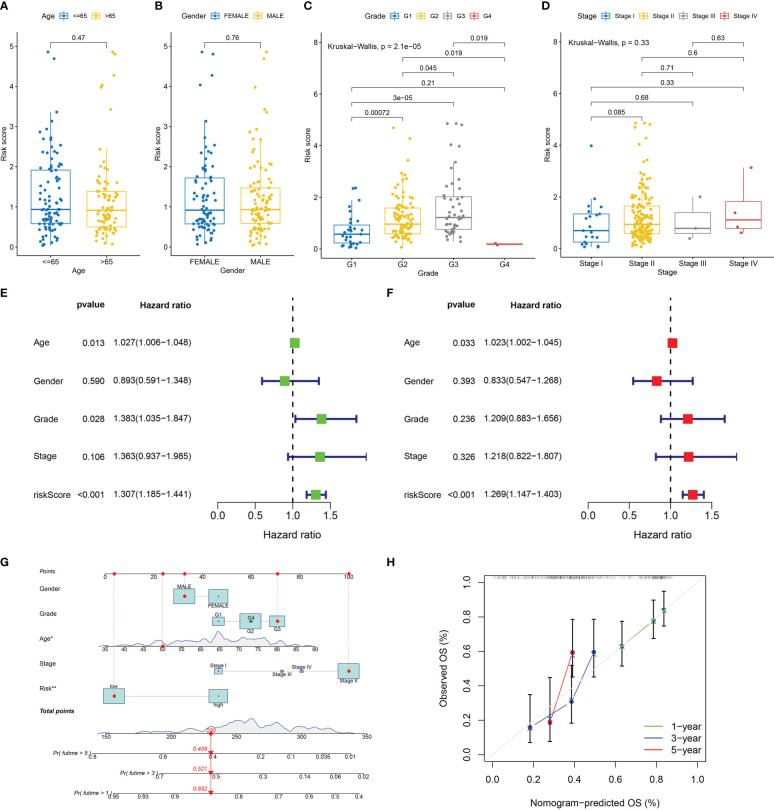
Clinicopathologic correlation, independent prognostic analysis, and nomogram model construction. Differences in risk score in age groups **(A)**, gender groups **(B)**, grade groups **(C)** and stage groups **(D)**. **(E)** The forest map of univariable Cox regression. **(F)** The forest map of multivariable Cox regression. **(G)** The nomogram prediction model based on risk score and clinicopathological characteristics. **(H)** Calibration curve of nomogram model of predicting 1, 3, 5 years survival rate.

The results showed that there was no difference in risk scores between different age and gender groups (**Figures 5A, B**). Risk scores were higher in higher pathological grade and the difference was statistically significant (**Figure 5C**). Risk scores increased gradually in the higher TNM stage, and the differences were close to statistical significance (**Figure 5D**). The expression of IL18 was associated with higher pathological grade and TNM stage (**Figure S1**). Univariate and multivariate Cox regression analyses confirmed that age and risk score independently acted as prognostic markers for pancreatic cancer (**Figures 5E, F**). Subsequently, utilizing clinicopathological parameters and risk score, we designed a nomogram model to predict the survival of patients with pancreatic cancer (**Figure 5G**). The calibration curve illustrated that the 1-, 3-, and 5- years survival rate predicted by the nomogram was close to the real survival, which signified that our nomogram model has outstanding predictive significance (**Figure 5H**).

### Gene set enrichment analyses

**Figure 6:**
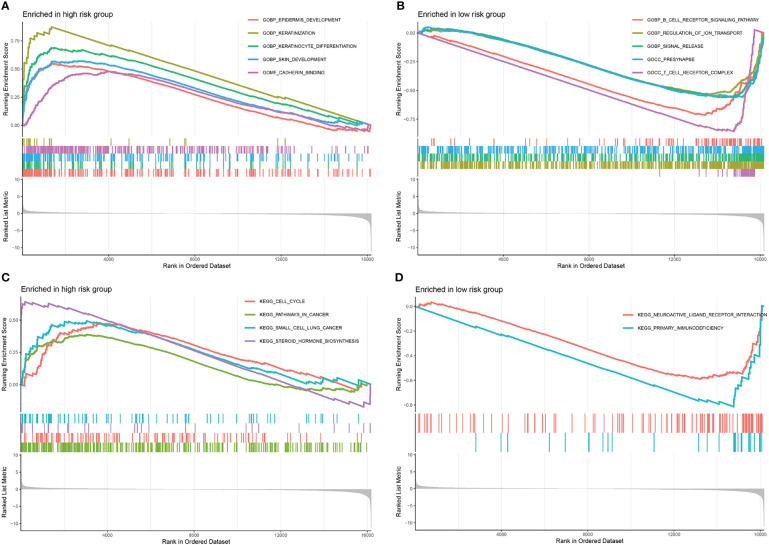
Gene set enrichment analyses. The top 5 significantly enriched pathways in gene set “c5.go.v7.5.1.symbols.gmt” of the high- **(A)** and low- **(B)** risk score groups. The significantly enriched pathways in gene set “c2.cp.kegg.v7.5.1.symbols.gmt” of the high- **(C)** and low- **(D)** risk score groups.

The GSEA was executed to investigate the variations between the high- and low-risk groups in terms of biological processes and metabolic pathways. In total, 274 pathways were considerably enriched in the gene set “c5.go.v7.5.1.symbols.gmt” (**Table S2**). In the high-risk group, the top 5 pathways with considerable enrichment were “epidermis development”, “keratinization”, “keratinocyte differentiation”, “skin development” and “cadherin binding” (**Figure 6A**). Additionally, the top 5 enriched pathways in the low-risk group were “B cell receptor signalling pathway”, “regulation of ion transport”, “signal release”, “presynapse” and “T cell receptor complex” (**Figure 6B**). In total, 6 pathways were considerably enriched in the gene set “c2.cp.kegg.v7.5.1.symbols.gmt”. The pathways with remarkable enrichment in the high-risk group were “cell cycle”, “pathways in cancer”, “small cell lung cancer” and “steroid hormone biosynthesis” (**Figure 6C**). Furthermore, the substantially enriched pathways in the low-risk were “neuroactive ligand-receptor interaction” and “primary immunodeficiency” (**Figure 6D**). According to the GSEA results, a remarkable enrichment in the high-risk group was mainly enriched in some pathways related to cancer, whereas the low-risk group was predominantly enriched in pathways linked to immune response.

### Immune analysis

**Figure 7:**
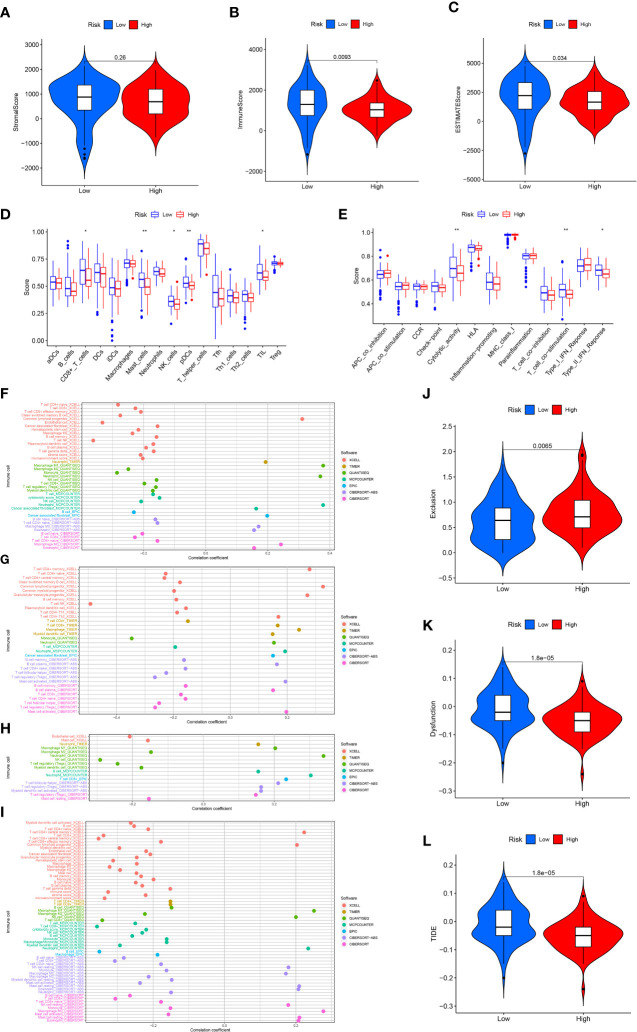
Immune analysis. Stromal **(A)**, immune **(B)** and ESTIMATE **(C)** score in the high- and low-risk score groups. 16 immune cell infiltration scores **(D)** and the activity score of 13 immune-related pathways **(E)** in the high- and low-risk score groups. Relationship between immune cell infiltration and risk score **(F)**, GSK3B **(G)**, IL18 **(H)** and VEGFA **(I)** expression level. T cell exclusion **(J)**, T cell dysfunction **(K)** and TIDE score **(L)** in the high- and low-risk score groups. *p<0.05;**p<0.01.

To analyze the link between immune infiltration and risk scores, we used several algorithms to contrast high- and low-risk groups. The “ESTIMATE” algorithm proved that stromal score had no significantly difference between high- and lowrisk groups (**Figures 7A**). The low-risk category has elevated immune and ESTIMATE scores in contrast with the high-risk population (**Figures 7B, C**). ssGSEA findings demonstrated that the low-risk category exhibited superior performance in immune cell infiltration and immune-related pathway in contrast with the high-risk subgroup, including NK cells, mast cells, CD8+ T cells, pDCs (Plasmacytoid dendritic cells), TIL (tumour Infiltrating lymphocyte), cytolytic activity, Type II IFN response, and T cell co-stimulation (**Figures 7D, E**). Immune cell infiltration analysis indicated that the risk score was significantly and inversely linked to naive CD4+ T cells, CD8+ T cells, macrophage M2, NK T cells, B cells, and T cell regulatory (Tregs) infiltration, significantly positively correlated with neutrophil and endothelial cell infiltration (**Figure 7F**). The expression of GSK3B was significantly inversely linked to the naive CD4+ T cells, memory B cells, NK T cells, and Tregs infiltration, and significantly positively linked to macrophage, neutrophil, cancer-associated fibroblast (CAFs), B cell plasma and activated mast cell infiltration (**Figure 7G**). The expression of IL18 was strongly and inversely linked to macrophage M2, mast cell and endothelial cell infiltration, and significantly positively correlated with neutrophil, macrophage M1, B cells, and CD8+ T cell infiltration (**Figure 7H**). The expression of VEGFA was significantly inversely linked to CD8+ T cell, B cell naïve, macrophage M2, endothelial cell infiltration, and significantly positively correlated with eosinophil and macrophage M0 infiltration (**Figure 7I**). Based on the calculated TIDE score, as well as T cell dysfunction and exclusion scores for each sample, we discovered that the high-risk population exhibited remarkably elevated T cell exclusion score (**Figure 7J**), whereas the low-risk population had significantly elevated T cell dysfunction score and TIDE score (**Figures 7K, L**). In addition, further analysis showed that the expression of GSK3B, IL18 and VEGFA were significantly associated with lower TIDE score (**Figure S2**). This illustrated that pancreatic cancer in the high-risk population had a greater likelihood of responding to immunotherapy as opposed to the low-risk population.

### Tumor mutation burden

**Figure 8:**
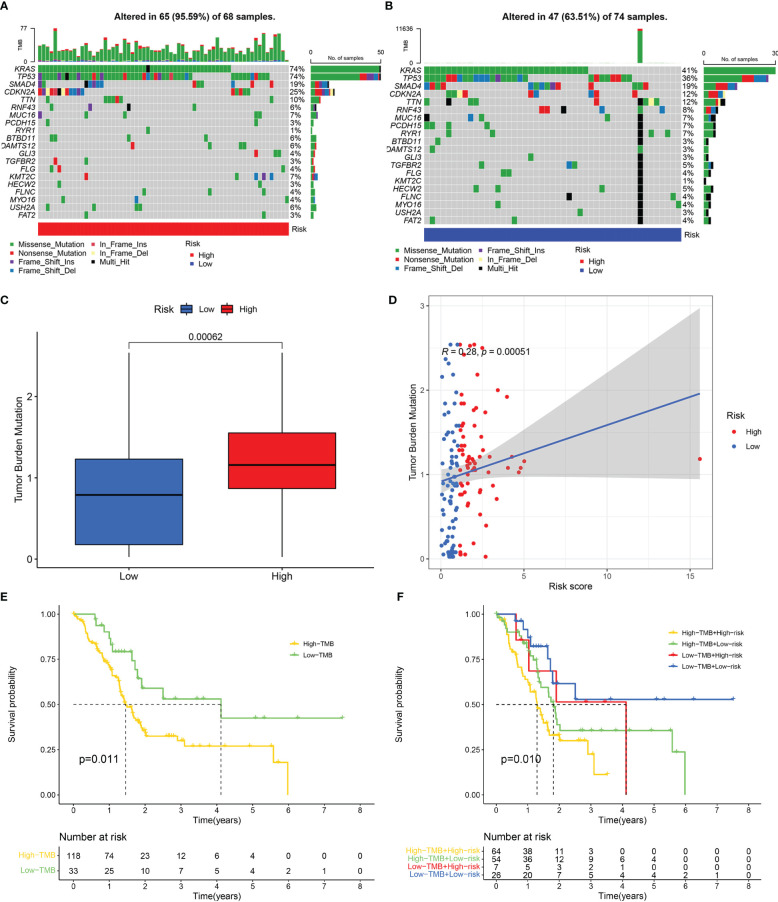
Mutation analysis. Genetic mutation landscape in the high- **(A)** and low- **(B)** risk score groups. **(C)** Tumour mutation burden (TMB) of the high- and low-risk score groups. **(D)** Correlation between TMB and risk score. **(E)** Kaplan-Meier curve of the high- and low-TMB categories. **(F)** Kaplan-Meier curve of the different TMB and risk score categories.

The “maftools” R package was utilized to analyze the mutation landscape in the high- and low-risk groups. A greater mutation frequency was seen in the high-risk group for the five most frequently mutated genes (TTN, CDKN2A, SMAD4, TP53, and KRAS) (**Figures 8A, B**). Previous studies indicated that activation mutations of the proto-oncogene KRAS and inactivation mutations of the tumour suppressor gene TP53, SMAD4 and CDKN2A were intimately linked to the occurrence, progression and dismal prognosis of pancreatic cancer (46, 47). TMB was depicted to be substantially enhanced in the high-risk group as per the Wilcoxon test (**Figure 8C**). Spearman test indicated a positive link between TMB and risk score (**Figure 8D**). We further analyzed the relationship between model genes and TMB, and the results showed that the expressions of GSK3B and VEGFA were significantly positively correlated with TMB (**Figure S3**). Survival analysis confirmed that TMB was linked to a worse outcome for patients with pancreatic cancer (**Figure 8E**). Accordingly, low risk and low TME were correlated with the best prognoses, whereas high risk and high TMB were linked to the worst prognoses (**Figure 8F**).

### Drug sensitivity

**Figure 9:**
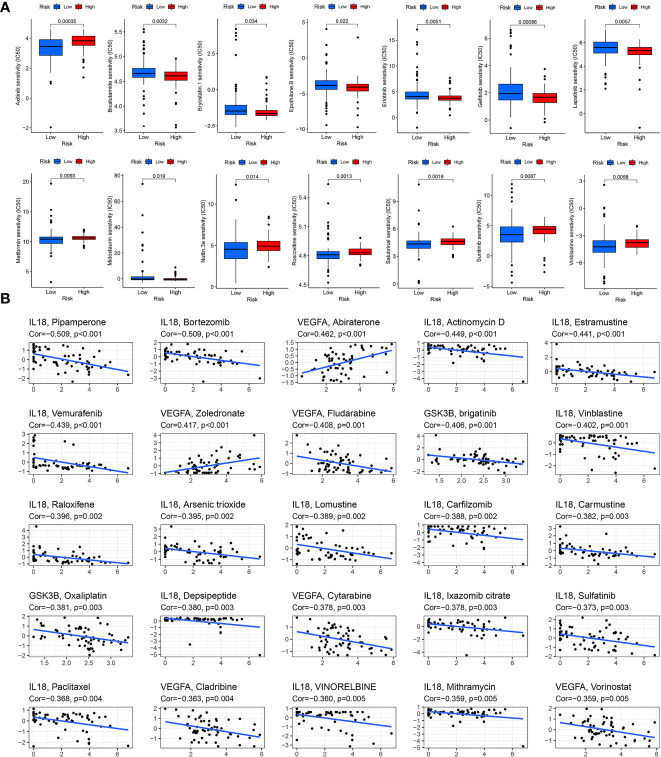
Drug sensitivity analysis. **(A)** Drug sensitivity in the high- and low-risk score groups. **(B)** Correlation between drug sensitivity and GSK3B, IL18 and VEGFA expression level.

Drug therapy is an important treatment for pancreatic cancer, especially for advanced pancreatic cancer. However, different patients have different sensitivity to different drugs. Therefore, it may be more effective and scientific to make individualized treatment plans for different patients. By applying the “pRRophetic” package in R software to predict drug sensitivity, we discovered that in high-risk group patients, 18 drugs (including BIBW2992, Bicalutamide, Gefitinib, Lapatinib, etc.) had significantly lower IC50 values, and in the low-risk group, 26 drugs (including Axitinib, Metformin, Roscovitine, Sunitinib, Vinblastine, etc.) had significantly lower IC50 values (**Table 1**). We selected 14 drugs shown in **Figure 9A**. Based on the relevant data from CellMiner database, we found that three genes in the model were associated with the sensitivity of 78 drugs (**Supplementary Table 3**), and the top 25 drugs with the most significant sensitivity were shown in **Figure 9B**. VEGFA expression was positively linked to the sensitivity of Abiraterone and Zoledronate, and inversely linked to Fludarabine, Cytarabine and Cladribine. GSK3B expression was inversely linked to Oxaliplatin and brigatinib. IL18 expression was negatively linked to Paclitaxel, VINORELBINE, Vinblastine and Sulfatinib.

**Table 1:** The sensitive drugs in the high- and low-risk score groups.

Group	Sensitive drugs
Low risk	ABT.263, ABT.888, AMG.706, ATRA, Axitinib, AZ628, AZD8055, BMS.536924, CEP.701, EHT.1864, GDC0941, KU.55933, Metformin, MK.2206, MS.275, Nutlin.3a, NVP.BEZ235, PD.173074, PD.0332991, PF.02341066, Roscovitine, Salubrinal, Sunitinib, TW.37, Vinblastine, Vorinostat.
High risk	A.443654, AUY922, BI.2536, BIBW2992, Bicalutamide, Bryostatin.1, Epothilone.B, Erlotinib, FTI.277, Gefitinib, GSK.650394, Lapatinib, LFM.A13, Midostaurin, NSC.87877, PLX4720, RDEA119, Thapsigargin.

### Gene expression verification and distribution analysis

**Figure 10:**
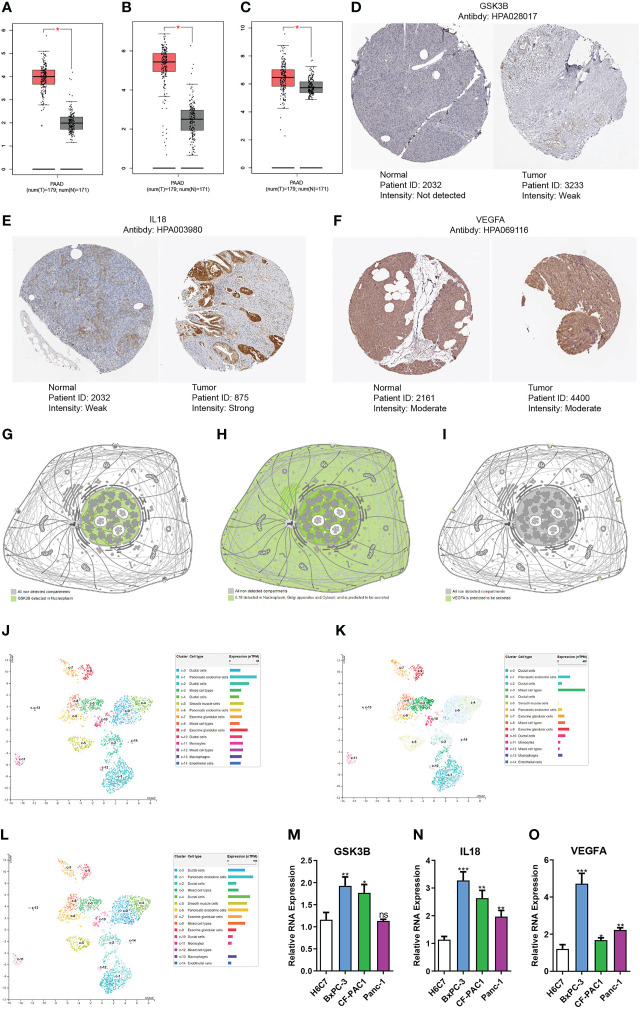
Gene expression verification and distribution analysis. Differences in the expression of GSK3B **(A)**, IL18 **(B)** and VEGFA **(C)** at RNA level between pancreatic normal tissues and tumor tissues based on GEPIA platform. Immunohistochemical images of GSK3B **(D)**, IL18 **(E)** and VEGFA **(F)** in pancreatic normal and tumor tissues. Distribution of GSK3B **(G)**, IL18 **(H)** and VEGFA **(I)** expression in different subcellular structures. Distribution of GSK3B **(J)**, IL18 **(K)** and VEGFA **(L)** expression in different cell types. Differences in the RNA expression of GSK3B **(M)**, IL18 **(N)** and VEGFA **(O)** between pancreatic normal cells and tumor cells based on RT-PCR. ns, no statistical significance; *p<0.05;**p<0.01;***p<0.001.

We used the GEPIA platform and discovered that GSK3B, IL18 and VEGFA RNA expression levels were elevated in tumors than in normal tissues (**Figures 10A-C**). qRT-PCR suggested that GSK3B, IL18 and VEGFA RNA expression level in tumor cells was remarkably higher in contrast with that in normal cells, in line with findings based on GEPIA platform (**Figures 10M-O**). Immunohistochemistry images derived from the HPA database showed that GSK3B and IL18 expression at protein level in tumour tissues was elevated in contrast with that in normal tissues, in line with findings of RNA expression levels (**Figures 10D, E**). However, there was no remarkably variations in the protein expression level of VEGFA in tumour tissues in contrast with normal tissues (**Figure 10F**). Subsequently, we additionally examined the distribution of the three genes’ expression in various subcellular structures and cell types by HPA database. GSK3B was detected in the nucleoplasm and mainly expressed in pancreatic endocrine, ductal and exocrine glandular cells (**Figures 10G, J**). IL18 was detected in the nucleoplasm, Golgi apparatus, and cytosol, and was also predicted to be secreted extracellular and mainly expressed in mixed cell types (**Figures 10H, K**). VEGFA was predicted to be secreted extracellular and predominantly expressed in pancreatic endocrine and ductal cells (**Figures 10I, L**). In addition, the single-cell dataset CAR001160 from the TISCH platform was utilized for further exploring the distribution of model genes in different cells in the TME of pancreatic cancer. Results showed that in the tumor microenvironment of pancreatic cancer, GSK3B was mainly distributed in endothelial cells, malignant cells and B cells, IL18 was mainly distributed in dendritic cells, monocytes/macrophages and malignant cells, and VEGFA was mainly distributed in malignant cells, monocytes/macrophages and ductal cells (**Figure S4**).

## Discussion

Pancreatic cancer is a very challenging malignant tumour with insidious onset, rapid progression and poor prognosis (48). The only current hope for curing pancreatic cancer is *via* major surgery. Unfortunately, by the time most patients are diagnosed, their chances of undergoing radical surgery have already been missed, and the effect of adjuvant therapy such as chemotherapy and radiotherapy on pancreatic cancer is not obvious (49). Finding effective new treatments for pancreatic cancer is crucial. In addition to antiangiogenic therapies and immunotherapies already in clinical practice, metabolic regulation is considered another promising approach for cancer treatment (50). Cancer is characterised in part by the metabolic reprogramming of its tissues. Compared with normal cells, pancreatic cancer cells undergo a series of metabolic alterations: (1) reprogramming the metabolism of intracellular nutrients, such as lipids, amino acids, and glucose; (2) enhancing nutrient supply through scavenging and recycling; (3) microenvironmental interactions involving metabolic processes and other components (51). These metabolic changes are conducive to the survival and division of pancreatic cancer cells in an environment of hypoxia and nutrient deprivation. The metabolism of tumors is often regulated by the neuroendocrine system, and abnormal neuroendocrine regulation may cause metabolic disorders. Studies showed that obesity could lead to abnormal adipose metabolism, chronic inflammation, insulin resistance, and hyperglycemia, and further affect the secretion of different hormones, growth factors, inflammatory cytokines, adipokines, and free fatty acids, which were considered to be the risk biomarkers for cancer morbidity and mortality (25, 52). Besides obesity, neuroendocrine diseases such as diabetes, depression, and anxiety can also enhance the risk of many malignancies and are linked to dismal prognoses (53, 54).

We developed an NMRGs-related prognostic model for pancreatic cancer using data in the TCGA database and by performing Cox and LASSO regression analyses, and the prognostic model was verified by GSE62452, GSE57495 and GSE28735 datasets. Unsupervised clustering, PCA and t-SNE analysis additionally proved the prognostic model’s reliability and consistency. The prognostic model included three genes: GSK3B, IL18 and VEGFA. GSK3B is a multifunctional serine/threonine kinase, which is implicated in various biological activities such as metabolism, cell cycle, DNA damage repair, cell proliferation, and apoptosis, and is associated with diabetes, tumors, psychiatric and neurodegenerative diseases (55–59). Darrington et al. (60) discovered that GSK3B expression level was elevated in prostate cancer (PCa) tissue in contrast with that in normal prostate tissue, and GSK3B inhibitors could reduce the growth of PCa cells. Mamaghani et al. (61) and Ougolkov et al. (62) illustrated that GSK3B expression was elevated in pancreatic cancer samples in contrast to normal pancreatic samples, which was congruent with our findings. Furthermore, the GSK3B inhibitor could suppress the survival and proliferation of pancreatic cancer cells by attenuating the activity of nuclear factor-kappaB (NF-κB). Studies indicated that GSK3B may play two roles in tumors: (1) promoting cancer through induced activation of NF-κB; (2) anti-cancer effect by preventing epithelial to mesenchymal transition (EMT) and metastasis (59, 63). Histone deacetylases (HDACs) could down-regulate the expression of E-cadherin in pancreatic cancer to promote EMT and metastasis, and HDACs inhibitors could suppress the proliferative and migratory capacities of pancreatic cancer cells (64). Edderkaoui et al. (63) found that metavert, a molecule that inhibits both GSK3B and HDACs activity, could significantly reduce tumour size, prevent metastasis, increase the killing of paclitaxel- and gemcitabine-resistant pancreatic cancer cells. IL-18 is a pro-inflammatory and immunomodulatory cytokine of the IL-1 family that is converted from an inactive precursor protein (pro-IL18) by caspase-1-induced cleavage of an N-terminal fragment and may have anti-cancer and oncogenic effects depending on the tissue and cellular environment (65, 66). Liu et al. (67) found that tongue squamous cell carcinoma may be prevented from advancing if IL18 is overexpressed since it may cause apoptosis and decrease the activity of the cells. However, Li et al. (68) illustrated that individuals with colorectal cancer who had an elevated blood IL-18 level had a worse prognosis. Kim et al. (69) found that IL18 could directly enhance the migratory ability of gastric cancer cells by filamentous-actin polymerization and tensin down-regulation. Guo et al. (70) illustrated that the expression level of IL18 was remarkably elevated in pancreatic cancer patient plasma in contrast with pancreatic benign tumors, pancreatitis, and healthy human plasma, elevated in pancreatic cancer tissues in contrast with normal tissues and was linked to a dismal prognosis of pancreatic cancer. This was consistent with our study results. VEGFA is a member of the vascular endothelial growth factor family, which participates in tumour angiogenesis and is intimately linked to tumour development and metastasis, and may be employed as a possible target for tumour therapy (71–73). Our study discovered that high expression of VEGFA was linked to the poor prognosis for pancreatic cancer.

TME is an intricate and comprehensive system in which tumour cells originate and live, which consists of tumour cells, stromal cells, immune cells, and extracellular matrix. TME is intimately linked to tumorigenesis, progression, and patient prognosis (74). GSEA results illustrated that pancreatic cancer in the low-risk group was predominantly enriched in immune-associated pathways. Furthermore, the “ESTIMATE” algorithm confirmed that patients in the low-risk subgroup had an elevated immune score. ssGSEA analysis further confirmed that immunocyte infiltration scores and immune-associated functional pathway scores were elevated in the low-risk group. Immune cell infiltration analysis confirmed that the infiltration degree of CD8+ T cells and NK cells was elevated in the low-risk group, whereas the infiltration degree of CAFs and neutrophil cells was elevated in the high-risk. CD8+ cytotoxic T cells perform an instrumental function in anti-tumour immunity by killing tumour cells, and can also inhibit angiogenesis by secreting interferon-gamma (IFN-γ), which is widely believed to be linked to improved prognosis of tumour patients (75, 76). Similar to CD8+ cytotoxic T cells, NK cells also perform an integral function in anti-tumour immunity, which can produce cytotoxic effects through effector cytokines, cytotoxic molecules and Fas pathway, and then kill tumour cells (74, 77). CAFs constitute the majority of stromal cells in the TME, including antigen-presenting CAFs (apCAFs), inflammatory CAFs (iCAFs), and Myofibrotic CAFs (myCAFs) and can reshape the extracellular matrix to enhance interstitial sclerosis, promote tumour invasion, induce chemotherapy resistance, inhibit antitumor T-cell response, and promote tumour growth (78). Tumour-associated neutrophils have been recognized as key players in malignant transformation, tumour progression, anti-tumour immunity and angiogenesis, and were associated with poor prognosis for advanced cancers and poor outcomes of immune checkpoint inhibitors therapy (79–81). CXCR2, the CXC receptor expressed by neutrophils, can bind with its ligand chemokine family (CXCL1, CXCL2, CXCL3, CXCL5, CXCL7, and CXCL8) to recruit neutrophils to the TME and participate in the mobilization of tumour-associated neutrophils (81, 82). Steele et al. (46) showed that inhibition of CXCR2 could slow tumour formation, prevent metastasis, and enhance the response to chemotherapy and immunotherapy in pancreatic cancer.

Immunotherapy is a promising treatment that has revealed considerable efficacy in numerous tumors, including melanoma, non-small cell lung cancer, renal cell carcinoma, hepatocellular carcinoma, and Hodgkin’s lymphoma (83–85). However, pancreatic cancer does not appear to be sensitive to immunotherapy, with a low overall response rate. The TIDE score was utilized to predict the link between immunotherapy and risk score, and the findings revealed that patients in the high-risk group exhibited a high likelihood of responding to immunotherapy. A greater TMB is often related to a greater rate of immunotherapy response, as evidenced by a series of studies (86, 87). TMB was considerably enhanced in the high-risk, as shown by our research, suggesting a positive link between risk score and TMB. Therefore, patients with pancreatic cancer in the high-risk group may have a greater sensitivity to immunotherapy, which is consistent with the result of TIDE score prediction. Besides surgery, chemotherapy is the main treatment for pancreatic cancer, especially for advanced pancreatic cancer. Our research revealed that the high- and low-risk groups differ remarkably in their susceptibility to certain small molecular drugs and chemotherapeutic medications. The level of IL18 expression is inversely linked to Paclitaxel sensitivity. GSK3B expression was inversely linked to the sensitivity of Oxaliplatin. Paclitaxel is often used together with gemcitabine to enhance the prognosis of patients with pancreatic cancer (88). Oxaliplatin is one of the chemotherapeutic drugs in FOLFIRINOX regimen (first-line treatment for pancreatic cancer) (89). Therefore, our risk score model is helpful to develop individualized treatment plans for patients with pancreatic cancer.

To our knowledge, this is the first study to use bioinformatics to comprehensively analyze the prognostic role of NMRGs in pancreatic cancer. Nonetheless, this investigation is not without its drawbacks. First, our data are from online databases TCGA and GEO, and the real prospective clinical cohorts are needed for further validation. Secondly, basic investigations still need to be conducted to better comprehend the function of NMRGs in the etiology and progression of pancreatic cancer.

## Conclusion

In summary, we established an NMRGs-related prognostic risk score model through the TCGA database, and the model was validated using GSE62452, GSE57495 and GSE28735 datasets. Unsupervised clustering analysis, PCA and t-SNE analysis further illustrated that the prognostic model has very good reliability. The prognostic risk score model contained three genes: GSK3B, IL18 and VEGFA, all of which were highly expressed in pancreatic cancer tissue and were associated with poor prognosis. In addition, our prognostic risk score model and model genes were closely linked to the immune infiltration microenvironment, TMB, and drug sensitivity, and can provide evidence for the treatment strategy of pancreatic cancer patients.

## Data availability statement

The datasets presented in this study can be found in online repositories. The names of the repository/repositories and accession number(s) can be found in the article/**Supplementary Material**.

## Author contributions

BiZ, QY, and BoZ conceptualized and designed the study. BiZ and BoZ collected the data from the database. BiZ and QY analyzed the data. QY and XC conducted the experiments. BiZ, SL, ZW, HL and FM wrote the article. XC and DS revised the manuscript. All authors contributed to the article and approved the submitted version.

## Supplementary material

Supplementary Figure 1 Clinicopathologic correlation. Differences of GSK3B expression in grade groups **(A)** and stage groups **(B)**. Differences of IL18 expression in grade groups **(C)** and stage groups **(D)**. Differences of VEGFA expression in grade groups **(E)** and stage groups **(F)**.

Supplementary Figure 2 The relationship between TIDE score and model genes. TIDE score in different GSK3B **(A)**, IL18 **(B)** and VEGFA **(C)** expression groups.

Supplementary Figure 3 The relationship between TMB and model genes. TMB in different GSK3B **(A)**, IL18 **(B)** and VEGFA **(C)** expression groups. Correlation between TMB and GSK3B **(D)**, IL18 **(E)** and VEGFA **(F)** expression.

Supplementary Figure 4 Single-cell analysis of model genes expression. **(A)** CAR001160 annotation of all cell types and percentage of each type. **(B, C)** Percentages and expressions of GSK3B, IL18 and VEGFA in different cells of pancreatic cancer tissues.

Supplementary Table 1 5552 differentially expressed genes between pancreatic tumour and normal tissues were identified by TCGA and GTEx databases, and 1173 neuroendocrine regulation-related genes and 1131 metabolism-related genes were obtained in the GeneCards platform.

Supplementary Table 2 Gene set enrichment analyses **(**GSEA) identified 274 remarkably enriched pathways between the high- and low-risk score groups.

Supplementary Table 3 Three genes in the model were associated with the sensitivity of 78 drugs based on the relevant data from CellMiner database.
