# Supplementary figures and images for: Characterization of neuroendocrine regulation- and metabolism-associated molecular features and prognostic indicators with aid to clinical chemotherapy and immunotherapy of patients with pancreatic cancer
Source: Front Endocrinol (Lausanne). 2023 Jan 20;13:1078424. doi: 10.3389/fendo.2022.1078424 (PMC9895410; doi:10.3389/fendo.2022.1078424)

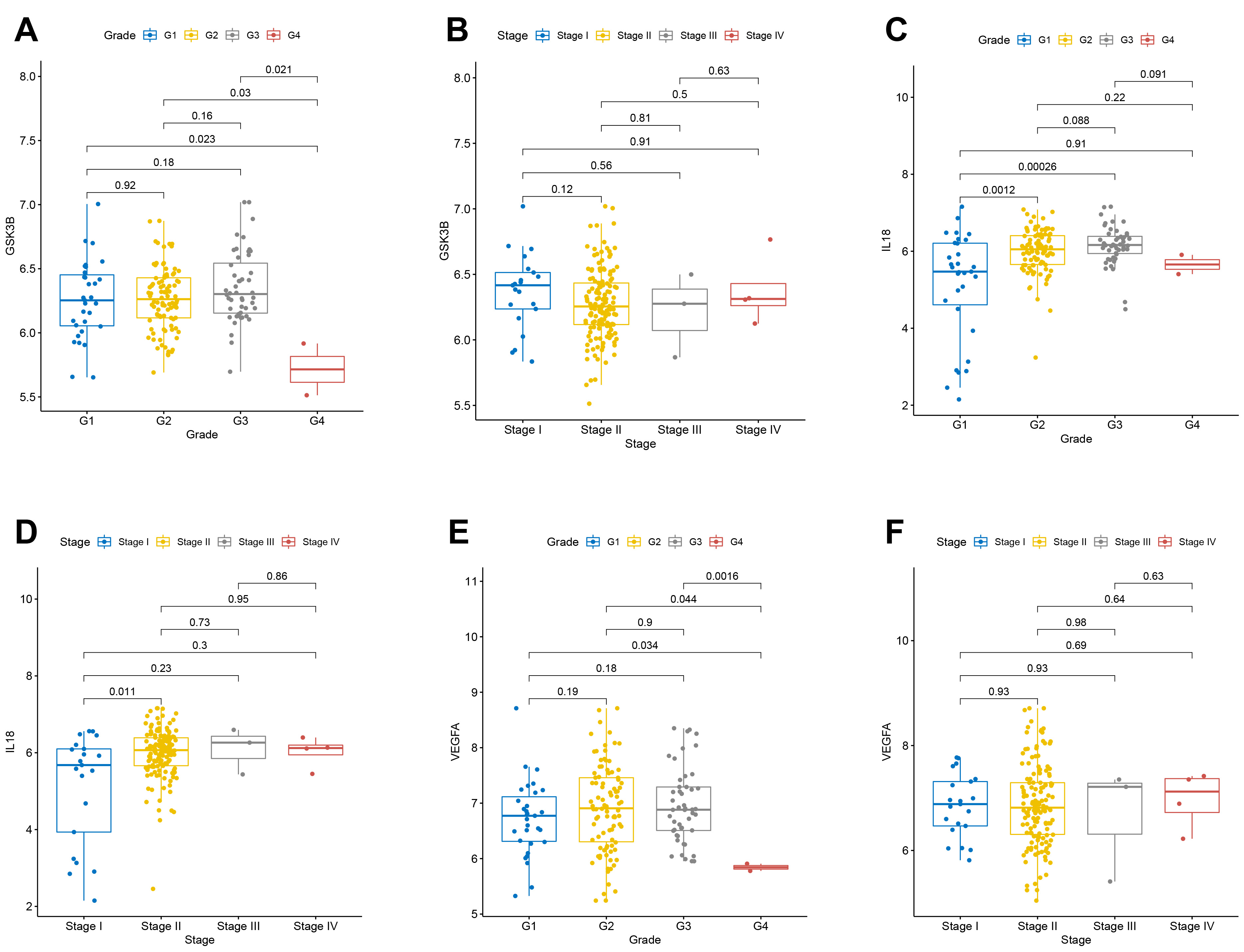

Supplement: Supplementary Figure 1 — Clinicopathologic correlation. Differences of GSK3B expression in grade groups (A) and stage groups (B). Differences of IL18 expression in grade groups (C) and stage groups (D). Differences of VEGFA expression in grade groups (E) and stage groups (F). [file Image_1.jpeg]

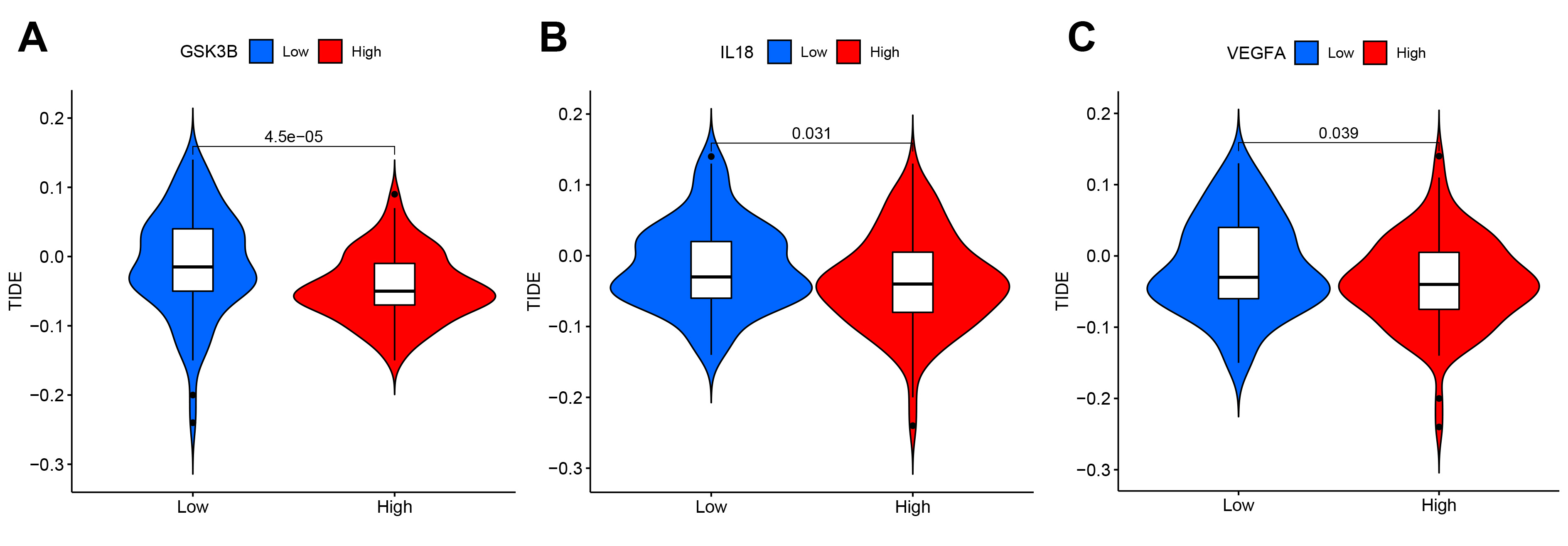

Supplement: Supplementary Figure 2 — The relationship between TIDE score and model genes. TIDE score in different GSK3B (A), IL18 (B) and VEGFA (C) expression groups. [file Image_2.jpeg]

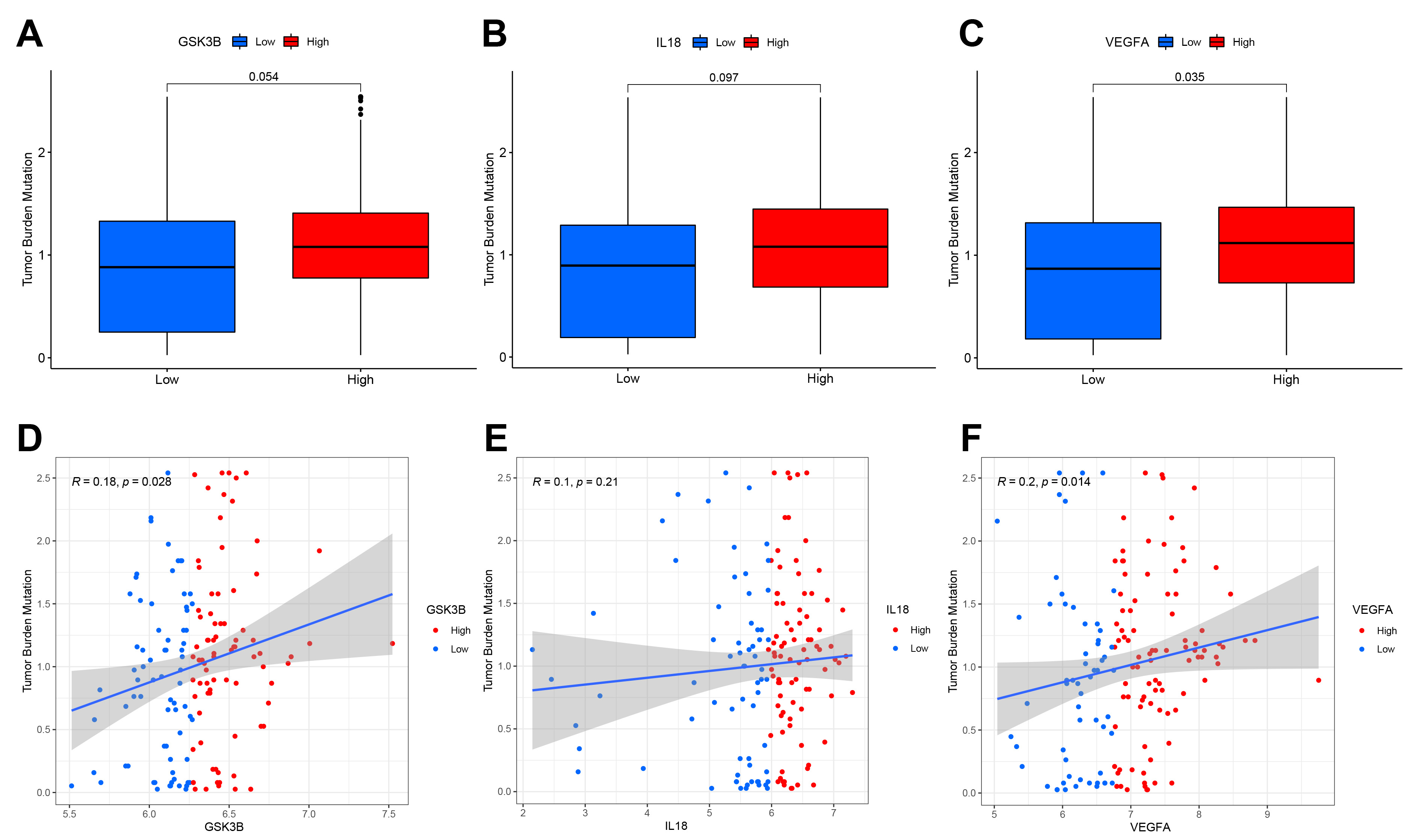

Supplement: Supplementary Figure 3 — The relationship between TMB and model genes. TMB in different GSK3B (A), IL18 (B) and VEGFA (C) expression groups. Correlation between TMB and GSK3B (D), IL18 (E) and VEGFA (F) expression. [file Image_3.jpeg]

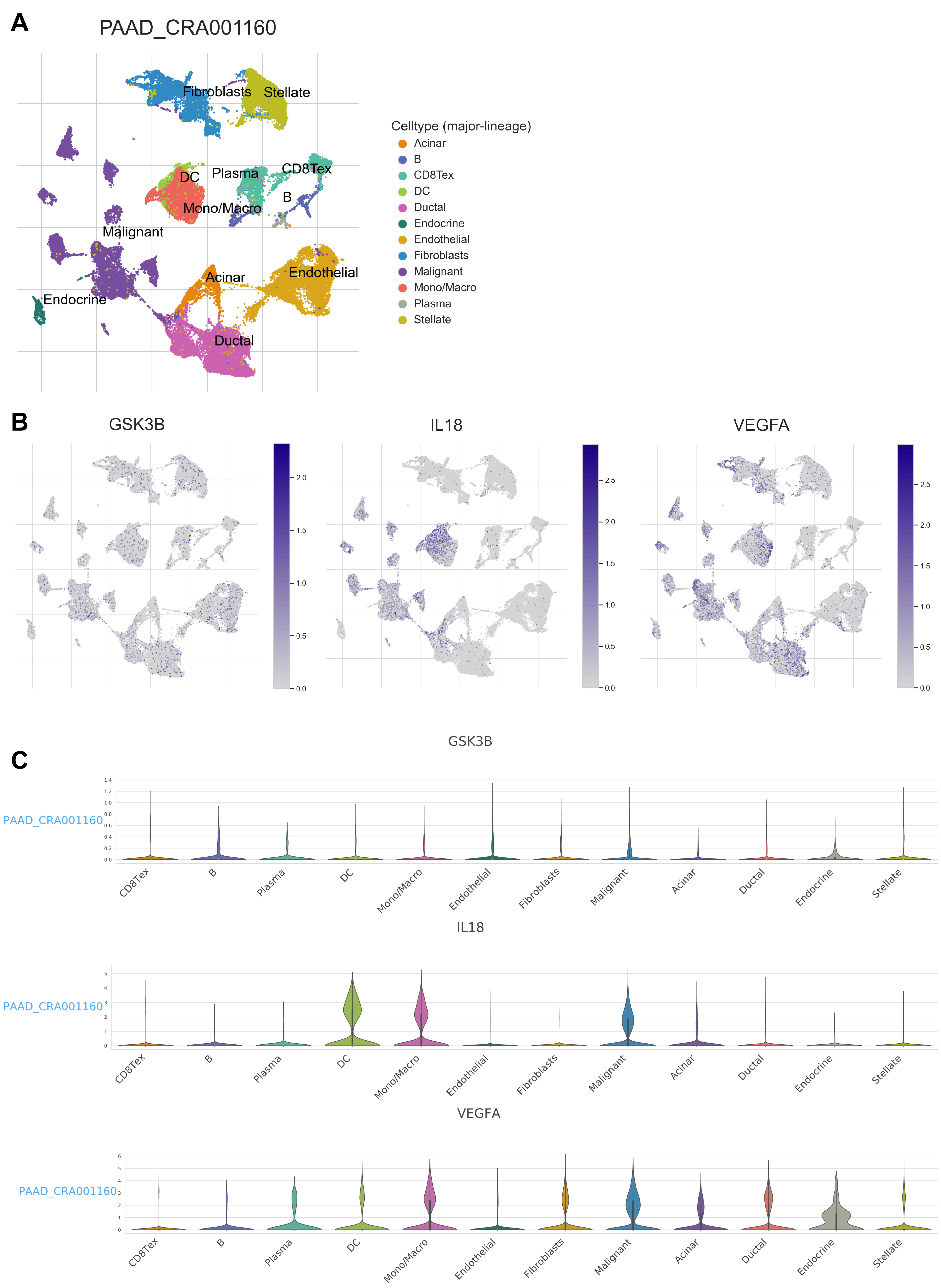

Supplement: Supplementary Figure 4 — Single-cell analysis of model genes expression. (A) CAR001160 annotation of all cell types and percentage of each type. (B, C) Percentages and expressions of GSK3B, IL18 and VEGFA in different cells of pancreatic cancer tissues. [file Image_4.jpeg]
